# Crescentic glomerular nephritis associated with rheumatoid arthritis: a case report

**DOI:** 10.1186/s13256-017-1346-8

**Published:** 2017-07-21

**Authors:** K. Balendran, L. D. S. U. Senarathne, R. D. Lanerolle

**Affiliations:** 1 University Medical Unit, National Hospital of Sri Lanka, Colombo 10, Colombo, Sri Lanka; 2 Department of Clinical Medicine, University of Colombo, Colombo, Sri Lanka

**Keywords:** Crescentic glomerular nephritis, Rheumatoid nephropathies, Rheumatoid arthritis

## Abstract

**Background:**

Rheumatoid arthritis is a systemic disorder where clinically significant renal involvement is relatively common. However, crescentic glomerular nephritis is a rarely described entity among the rheumatoid nephropathies. We report a case of a patient with rheumatoid arthritis presenting with antineutrophil cytoplasmic antibody-negative crescentic glomerular nephritis.

**Case presentation:**

A 54-year-old Sri Lankan woman who had recently been diagnosed with rheumatoid arthritis was being treated with methotrexate 10 mg weekly and infrequent nonsteroidal anti-inflammatory drugs. She presented to our hospital with worsening generalized body swelling and oliguria of 1 month’s duration. Her physical examination revealed that she had bilateral pitting leg edema and periorbital edema. She was not pale or icteric. She had evidence of mild synovitis of the small joints of the hand bilaterally with no deformities. No evidence of systemic vasculitis was seen. Her blood pressure was 170/100 mmHg, and her jugular venous pressure was elevated to 7 cm with an undisplaced cardiac apex. Her urine full report revealed 2+ proteinuria with active sediment (dysmorphic red blood cells [17%] and granular casts). Her 24-hour urinary protein excretion was 2 g. Her serum creatinine level was 388 μmol/L. Abdominal ultrasound revealed normal-sized kidneys with acute parenchymal changes and mild ascites. Her renal biopsy showed renal parenchyma containing 20 glomeruli showing diffuse proliferative glomerular nephritis, with 14 of 20 glomeruli showing cellular crescents, and the result of Congo red staining was negative. Her rheumatoid factor was positive with a high titer (120 IU/ml), but results for antinuclear antibody, double-stranded deoxyribonucleic acid, and antineutrophil cytoplasmic antibody (perinuclear and cytoplasmic) were negative. Antistreptolysin O titer <200 U/ml and cryoglobulins were not detected. The results of her hepatitis serology, retroviral screening, and malignancy screening were negative. Her erythrocyte sedimentation rate was 110 mm in the first hour, and her C-reactive protein level was 45 mg/dl. Her liver profile showed hypoalbuminemia of 28 g/dl. She was treated with immunomodulators and had a good recovery of her renal function.

**Conclusions:**

This case illustrates a rare presentation of antineutrophil cytoplasmic antibody-negative crescentic glomerular nephritis in a patient with rheumatoid arthritis, awareness of which would facilitate early appropriate investigations and treatment.

## Background

Rheumatoid arthritis is a systemic disorder that primarily affects the joints, but renal involvement is relatively common and clinically significant because it worsens the course and mortality of the primary disease [1]. Renal involvement in rheumatoid arthritis includes secondary amyloidosis, nephrotoxicity of the drugs used for treatment, and rheumatoid nephropathy as extra-articular manifestations [1–5]. In rheumatoid nephropathies, mesangial glomerular nephritis is the most frequent histological lesion, followed by minimal change glomerulopathy, membranous glomerulopathy, and crescentic glomerular nephritis [1, 2, 6–10]. Crescentic glomerular nephritis is rare, and more than 50% of the patients have features of systemic vasculitis, with almost all having perinuclear antineutrophil cytoplasmic antibody positivity [9, 10]. Only one case of rheumatoid arthritis-associated antineutrophil cytoplasmic antibody (ANCA)-negative crescentic glomerular nephritis has been reported to date [10]. We report a case of a patient with rheumatoid arthritis who presented with ANCA-negative crescentic glomerular nephritis without frank systemic vasculitis.

## Case presentation

A 54-year-old Sri Lankan woman who had recently been diagnosed with rheumatoid arthritis presented to our hospital with worsening bilatateral leg swelling and facial puffiness of 1 month’s duration, accompanied by oliguria. She had no frothy urine or hematuria. She did not have exertional breathlessness or orthopnea, and she had no history suggestive of a cardiac or hepatic cause of edema. She was not diabetic or hypertensive. Her rheumatoid arthritis had been diagnosed 8 months earlier, when she presented with bilateral symmetrical polyarthritis involving the small joints of her hands with significant morning stiffness of 2 hours’ duration. Her rheumatoid factor was positive at a high titer. She was commenced on methotrexate 10 mg weekly and infrequent nonsteroidal anti-inflammatory drugs, with good symptom control achieved.

Her physical examination revealed that she had bilateral pitting leg edema and periorbital edema. She was not pale or icteric. No malar rash, vasculitic rash, or distal gangrene was seen. She had evidence of mild synovitis of the small joints of the hands bilaterally with no deformities. No generalized lymphadenopathy or hepatosplenomegaly was noted. Her pulse rate was 90 beats per minute; her blood pressure was 170/100 mmHg; and her jugular venous pressure was elevated to 7 cm with an undisplaced cardiac apex and normal heart sounds. A fundus examination did not reveal papilledema. Her lungs were clear with equal breath sounds bilaterally. The results of the rest of the examination were normal. Her laboratory investigation results are provided in Table 1.

**Table 1:** Patient’s laboratory examination results

Examinations	Results		
Urine full report	Protein 2+ Pus cells: 5–10/HPF Red blood cells: moderately full field Few granular casts present		
Twenty-four-hour urinary protein excretion	2 g/24 hours		
Dysmorphic red blood cells	17%		
Serum creatinine	2 months before presentation: 88 μmol/L	On admission: 389 μmol/L	Posttreatment: 110 μmol/L
Serum electrolytes	Sodium: 138 mmol/L		
	Potassium: 4.1 mmol/L		
Inflammatory markers	ESR: 110 mm in first hour		
	CRP: 45 mg/dl		
Fasting blood sugar	95 mg/dl		
Abdominal ultrasound	Normal-sized kidneys with acute parenchymal changes and mild ascites No organomegaly		
Renal biopsy	Renal parenchyma containing 20 glomeruli showing diffuse proliferative glomerular nephritis, with 14 of 20 glomeruli showing cellular crescents, and result of Congo red staining was negative Immune fixation not done, owing to its unavailability		
Immunological markers	Rheumatoid factor: high titer (120 IU/ml)		
	ANA- and dsDNA-negative		
	ANCA-negative (perinuclear and cytoplasmic)		
	Complement C3: 98 mg/dl (normal range 90–180)		
	Complement C4: 21 mg/dl (normal range 10–40)		
	ASOT: <200 U/ml		
	Cryoglobulins: not detected		
Other secondary causes screening	Hepatitis B surface antigen-negative		
	Hepatitis C antibody-negative		
	HIV-1- and HIV-2-negative		
	Malignancy screening: negative		
	Screening tests done: chest x-ray, ultrasound thyroid, abdomen and pelvis, mammogram, gastrointestinal endoscopy		
Liver profile	Albumin: 28 g/L Globulin: 33 g/L		
	Transaminases and bilirubin: normal		

*Abbreviations: ANA* Antinuclear antibody, *ANCA* Antineutrophil cytoplasmic antibody, *ASOT* Antistreptolysin O titer, *CRP* C-reactive protein, *dsDNA* Double-stranded deoxyribonucleic acid, *ESR* Erythrocyte sedimentation rate, *HIV* Human immunodeficiency virus, *HPF* High-power field

A diagnosis of crescentic glomerular nephritis was made. The patient was started on atorvastatin, enalapril, and diuretics. Intravenous methylprednisolone 1 g was given for 3 consecutive days, followed by 1 mg/kg oral prednisolone. She was started on intravenous cyclophosphamide 500 mg every 2 weeks for a total of six doses. She gradually had increasing urine output and was symptomatically better, with improving renal function. Her serum creatinine level was 110 μmol/L at her last clinic visit after 3 months of treatment.

## Discussion

A middle-aged woman with seropositive rheumatoid arthritis presented to our hospital with progressively worsening generalized edema with features of intravascular volume overload. Investigations revealed a subnephrotic range of proteinuria with active sediment and impaired renal function with histological evidence of crescentic glomerular nephritis.

Because crescentic glomerular nephritis is a rare entity in rheumatoid nephropathy, we looked for other causes of crescentic glomerular nephritis. Our patient did not have clinical features of systemic vasculitis. Her antibody profile was negative for systemic lupus erythematosus, medium-vessel vasculitis, and cryoglobulinemia. Also, the result of her solid organ malignancy screening was negative. She was treated with methylprednisolone and cyclophosphamide pulses and had good recovery of her renal function.

Crescentic glomerulonephritis is a rarely described entity [11–13]. These patients generally present with microscopic hematuria, proteinuria, and renal impairment, as seen in our patient. It is usually associated with seropositive erosive disease with a median duration of arthritis of 12 years (range 1–25 years) [11]. However, our patient presented within 1 year of receiving her seropositive rheumatoid arthritis diagnosis and did not have erosive arthritis. To the best of our knowledge, only one case of rheumatoid arthritis-associated ANCA-negative crescentic glomerular nephritis has been reported to date [10]. Crescentic glomerular nephritis needs aggressive treatment with immunomodulators, including intravenous methylprednisolone pulses and cyclophosphamide [14].

## Conclusions

Crescentic glomerular nephritis without systemic vasculitis as an extra-articular manifestation in rheumatoid arthritis is rare but has severe clinical manifestations. Early diagnosis and treatment are vital.

## Abbreviations

ANA: Antinuclear antibody
ANCA: Antineutrophil cytoplasmic antibody
ASOT: Antistreptolysin O titer
CRP: C-reactive protein
dsDNA: Double-stranded DNA
ESR: Erythrocyte sedimentation rate
HPF: High-power field
